# Interleukin-17 Reduces βENaC via MAPK Signaling in Vascular Smooth Muscle Cells

**DOI:** 10.3390/ijms21082953

**Published:** 2020-04-22

**Authors:** Jeremy W. Duncan, Joey P. Granger, Michael J. Ryan, Heather A. Drummond

**Affiliations:** Department of Physiology and Biophysics, University of Mississippi Medical Center, Jackson, MS 39206, USA; jduncan2@umc.edu (J.W.D.); jgranger@umc.edu (J.P.G.); mjryan@umc.edu (M.J.R.)

**Keywords:** IL-17, degenerins, beta-ENaC, VSMCs, p38MAPK, JNK, NFκB

## Abstract

Degenerin proteins, such as the beta epithelial Na^+^ channel (βENaC), are essential in the intracellular signaling of pressure-induced constriction, an important vascular smooth muscle cell (VSMC) function. While certain cytokines reduce ENaC protein in epithelial tissue, it is unknown if interleukin-17 (IL-17), a potent pro-inflammatory cytokine, directly mediates changes in membrane-associated βENaC in VSMCs. Therefore, we tested the hypothesis that exposure to IL-17 reduces βENaC in VSMCs through canonical mitogen-activated protein kinase (MAPK) signaling pathways. We treated cultured rat VSMCs (A10 cell line) with IL-17 (1–100 ng/mL) for 15 min to 16 h and measured expression of βENaC, p38MAPK, c-jun kinase (JNK), and nuclear factor kappa-light-chain-enhancer of activated B cells (NFκB). IL-17 reduced βENaC protein expression in a concentration-dependent fashion and increased phosphorylation of p38MAPK by 15 min and JNK by 8 h. NFκB was unaffected by IL-17 in VSMCs. IL-17 treatment reduced VSMC viability but had no effect on cell death. To determine the underlying signaling pathway involved in this response, VSMCs were treated before and during IL-17 exposure with p38MAPK or JNK inhibitors. We found that JNK blockade prevented IL-17-mediated βENaC protein suppression. These data demonstrate that the pro-inflammatory cytokine IL-17 regulates VSMC βENaC via canonical MAPK signaling pathways, raising the possibility that βENaC-mediated loss of VSMC function may occur in inflammatory disorders.

## 1. Introduction

Vascular smooth muscle cells (VSMCs) are an integral component of vascular blood flow regulation. Pressure-induced constriction, also known as myogenic constriction, is an inherent response of certain small arteries and arterioles, mediated by VSMCs, and a mechanism of local blood flow autoregulation. The response is initiated when increases in intraluminal pressure cause vessel distention and elongation of circumferentially oriented VSMCs [1]. While numerous molecules contribute to transduction of stretch-induced VSMC contraction, our laboratory has focused on the importance of degenerin proteins in initiation of this response [1,2,3,4,5,6,7,8,9,10,11,12,13,14,15,16,17,18].

Degenerin proteins are an evolutionarily conserved family of cation channels, where many members function as sensors [4,6,19,20]. Epithelial Na^+^ channel (ENaC) proteins are members of this family. The concept that ENaC function is limited to canonical αENaC channels mediating Na^+^ transport in epithelial tissue is evolving. ENaC channel expression is not limited to epithelial tissue and individual subunits are also capable of forming current-generating channels [20,21,22,23,24]. ENaC proteins are also expressed in VSMCs. βENaC is the most abundantly expressed of the three subunits and plays a critical role in mediating pressure-induced changes in vascular tone in small cerebral arteries, renal interlobar arteries, and renal afferent arterioles [5,8,17].

Epithelial ENaC expression is regulated by many factors, including pro-inflammatory cytokines and interleukins [24,25,26,27,28,29]. Interleukin (IL)-1β, IL-4, and IL-13 inhibition of ENaC expression and function is dependent on extracellular signal-related kinase (ERK)1/2 and p38 mitogen-activated protein kinase (p38MAPK) signaling pathways [25,26,29,30]. IL-1β decreases the expression of αENaC via p38-MAPK in AT2 cells, while IL-4 reduces the expression of γ and β transcripts in human bronchi epithelial cells [28,29]. A recent study from our laboratory shows that the pro-inflammatory cytokine tumor necrosis factor-alpha (TNF-α) inhibits βENaC in VSMCs; however, it is unknown if IL-17 acts in a similar manner. IL-17 is the founding member of a subclass of potent inflammatory cytokines and is implicated in a number of pro-inflammatory disorders [31,32].

Pro-inflammatory cytokines are linked to vascular dysfunction through a number of mechanisms, including cytokine-mediated oxidative stress, apoptosis, metabolic effects, cell signaling pathways, and direct regulation of vascular proteins [31,33,34,35,36,37,38,39,40,41,42,43,44]. However, it is unclear which inflammatory factors are important drivers of potential VSMC impairment by changes in βENaC. Therefore, the goal of this study was to determine whether IL-17, one of the most well characterized cytokines in inflammatory disorders, regulates βENaC protein via MAPK signaling in VSMCs. The results of this study demonstrate that IL-17 exposure inhibits VSMC βENaC expression through canonical MAPK signaling pathways. These findings broaden our understanding of the regulation and potential importance of an ion channel once thought to have a markedly limited role and expression pattern.

## 2. Methods

### 2.1. Cell Culture

The A10 VSMC cell line (rat aortic/thoracic VSMCs, American Type Cell Culture, Manassas, Virginia, USA) was utilized for cell studies. Cells were grown at 37 °C with 5% CO_2_ in DMEM (Sigma–Aldrich, St. Louis, MO, USA) supplemented with 10% FBS and 1% penicillin/streptomycin on T-75 flasks. Cells were seeded at a density of 1 × 10^6^/mL onto 6-well plates or 120 mm dishes.

### 2.2. Cell Treatment

To determine direct effect of IL-17 on VSMC βENaC expression, cell culture media was supplemented with 0–100 ng/mL recombinant rat IL-17 (Cat # 510-RT-010; R&D Systems, Minneapolis, MN, USA) for 16 h. For phosphorylation studies, cells were treated with IL-17 (100 ng/mL) for 15 m, 2 h, or 8 h to measure p38MAPK and c-jun kinase (JNK) activation, or 16 h to measure nuclear factor kappa-chain-light-enhancer of B cells (NFκB) activation. To examine the importance of kinase signaling pathways, cells were pre-incubated with p38MAPK (10 µM) or JNK (50 µM) inhibitors (Tocris, SB 203580, #1202; and SP 600125, #1496, Minneapolis, MN, USA, respectively) for 1 h, then exposed to 100 ng/mL IL-17 plus MAPK inhibitors for 16 h.

### 2.3. Protein Isolation

To determine the effect IL-17 on βENaC expression, total membrane protein was isolated using the Membrane Protein Extraction Kit (Biovision, Cat# K268, Milpitas, CA, USA). Membrane fractions were sonicated and collected by centrifugation at 15,000 RPM for 45 min at 4 °C and stored at −20 °C until further analysis. To determine the effect of IL-17 on p38/JNK/NFκB phosphorylation, RIPA buffer supplemented with protease and kinase inhibitors (Santa Cruz Biotechnology, Santa Cruz, CA, USA) was used. Cell lysates were centrifuged at 15,000 RPM for 45 min at 4 °C and stored at −20 °C until further analysis. Protein content was determined by BCA analysis according to the manufacturer’s instructions (Pierce BCA Protein Assay Kit, Waltham, MA, USA).

### 2.4. Western Blot on A10 Protein Isolates

Protein samples (15–25 µg) were separated using 10% (βENaC), 4%–20% gradient (NFκB) or 12.5% (p38MAPK/JNK) Tris-HCl gels (BioRad, Hercules, CA, USA) for 1.5 h at 120 V. Molecular weight was estimated by Precision Plus Protein Standards (BioRad). Separated proteins were transferred to nitrocellulose membranes for 1.5 h at 60 V and membranes were blocked for 1 h at room temperature in Odyssey Blocking Buffer (Li-Cor Biosciences, Lincoln, NE, USA). Blots were incubated with rabbit anti-βENaC_C-term_ (1:2500) for 48–72 h at 4 °C [5,9,19] (see Supplementary Materials). The rabbit antibody is targeted to the extreme COOH terminus of mouse βENaC and commercially generated for our laboratory and tested for specificity using multiple approaches including expression of βENaC in heterologous cell lines, βENaC mutant (knockdown) renal sections and isolated VSMCs, as well as siRNA and dominant-negative gene silencing approaches. The specificity of this antibody has been characterized in several publications [5,9,19]. Mouse anti-β-actin (1:10,000, Ab6276, Abcam, Cambridge, MA) served as a loading control for assessing changes in βENaC due to variability in membrane protein loading [5,9,17,19]. Separate membranes were probed with the following antibody combinations: rabbit anti-p38MAPK/mouse anti-phospho p38MAPK, rabbit anti-JNK/mouse anti-phospho JNK, and rabbit anti-phospho NFκB/mouse anti-NFκB (1:1000; Cell Signaling Technology, Beverly, MA, USA) overnight at 4 °C. Membranes were rinsed with PBS + 0.1% Tween 20 and incubated with IR700-conjugated goat anti-rabbit and IR800-conjugated goat anti-mouse IgG (1:10,000; Li-Cor) for 1 h at room temperature, then rinsed. Membranes were visualized using an Odyssey Infrared Imaging System (Li-Cor). Lanes with artifacts, inefficient loading/transferring, or identified as outliers by ROUT (Prism, San Diego, CA, USA) were excluded from analysis. All experimental samples were normalized to the control sample(s) of each trial and expressed as a percent of the respective control. Each experiment contained 2 or more trials and was expressed as a percent of control.

### 2.5. Live/Dead Viability/Cytotoxicity Assay

The LIVE/DEAD Viability/Cytotoxicity kit (Invitrogen Molecular Probes, Carlsbad, CA, USA) was used to determine the effect of IL-17 on VSMC viability. Live and dead cells were simultaneously detected by using two fluorescence probes: Calcein-AM, a probe for intracellular esterase activity, labels live cells, while ethidium homodimer-1 (ED-1), a probe for loss of plasma membrane integrity, labels dead cells. VSMC monolayers were cultured in 96-well plates and treated for 16 h with IL-17 (1–100 ng/mL). Untreated control cells and cells treated with 70% methanol served as positive controls when assaying live and dead cells, respectively. Following treatment, cells were labeled with Calcein-AM and ethidium homodimer-1, according to manufacturer’s protocol. Fluorescence was quantified on a microplate reader. The live:dead ratio was calculated by dividing the fluorescence of Calcein-AM by ED-1 and normalizing as percent of control cells.

### 2.6. Statistical Analysis.

All data were analyzed using a one-way ANOVA, followed by Holm–Sidak post hoc test. A trend analysis was used to determine concentration/time-dependent responses. All statistical analyses were performed using Prism software (GraphPad, San Diego, CA, USA). All data are presented as mean ± standard error of the mean (SEM). Certain p values are provided to demonstrate level of confidence.

## 3. Results

### 3.1. IL-17 Reduces βENaC Expression in a Concentration-Dependent Fashion in Cultured VSMCs

**Figure 1:**
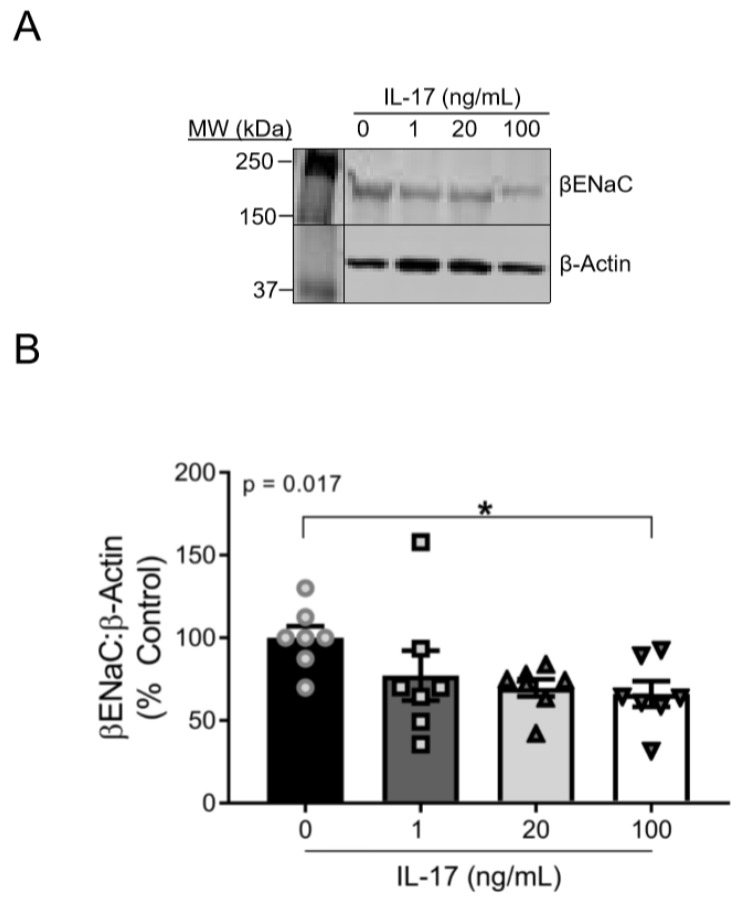
IL-17 reduces the protein expression of βENaC in cultured VSMCs in a concentration-dependent fashion. (**A**) Representative immunoblots showing βENaC and β-actin. (**B**) Quantification of βENaC following 16 h treatment of IL-17 at the following concentrations: 0, 1, 20, and 100 ng/mL (*n* = 7/group). Comparisons made by one-way ANOVA. The *p* value represents a post hoc analysis test for linear trend. Data are presented as mean ± SEM. * Significantly different from control at *p* < 0.05.

Supplemental IL-17 (1–100 ng/mL) induced a concentration-dependent reduction in βENaC expression (linear slope of the relationship of IL-17 concentration and βENaC = −11.24 (% increase in βENaC:IL-17 ng/mL; *p* = 0.015)) after 16 h of treatment. While a concentration-dependent effect of IL-17 on βENaC was present, the change in βENaC from 20–100 ng/mL was modest. βENaC protein was reduced in 100 ng/mL IL-17-treated cells to 65% ± 8% of control cells (100 ± 8%; *p* = 0.049). Representative blots for βENaC and β-actin are shown in Figure 1A and group data in Figure 1B.

### 3.2. Reduction in βENaC by IL-17 Is Not Associated with Cell Death

**Figure 2:**
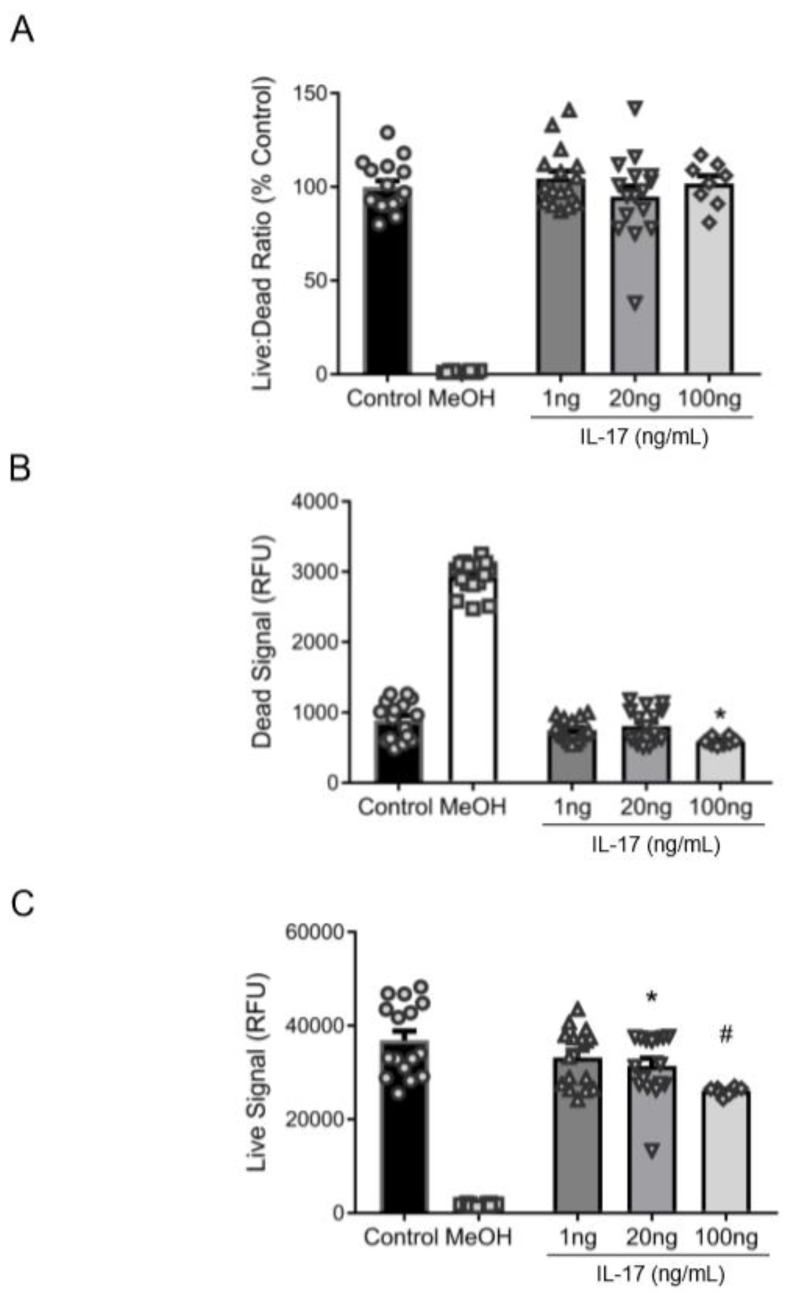
IL-17 does not induce cell death in VSMCs. To determine whether a reduction in βENaC by IL-17 was associated with cell death or reduced viability, cultured VSMCs were treated with IL-17 for 16 h to determine the amount of live and dead cells. Control cells (*n* = 16) and cells treated with IL-17 at 1 (*n* = 16), 20 (*n* = 16), and 100 (*n* = 8) ng/mL were examined. MeOH (*n* = 16) and Calcein-AM (*n* = 16) was used as negative and positive controls for live and dead signals, respectively. (**A**) The ratio of live:dead cells expressed as a percent of control. (**B**) Quantification of live cells following IL-17 treatment. (**C**) Quantification of dead cells following IL-17 treatment. Comparisons were made by one-way ANOVA, followed by the Holms–Sidak post hoc test. All data are presented as mean ± SEM. * Significantly different from control at *p* < 0.05. # Significantly different from control at *p* < 0.001.

To determine whether the IL-17-mediated decrease in βENaC was attributable to cell death, we examined cell viability in cultured VSMCs. While IL-17 treatment did not alter the live:dead fluorescence ratio (Figure 2A), 20–100 ng/mL reduced the Calcein-AM fluorescence (“viable” signal) (Figure 2B), indicating that IL-17 impairs cell viability/proliferation. The ethidium homodimer-1 fluorescence (“dead” signal) was reduced at 100 ng/mL, suggesting that high concentrations of IL-17 were protective and did not cause cell death (Figure 2C). These data suggest that IL-17 treatment reduced VSMC viability but did not increase cell death, indicating the IL-17-mediated reduction in VSMC βENaC is not due to cell death.

### 3.3. IL-17 Induces the Phosphorylation of p38MAPK and JNK, but Not NFκB

**Figure 3:**
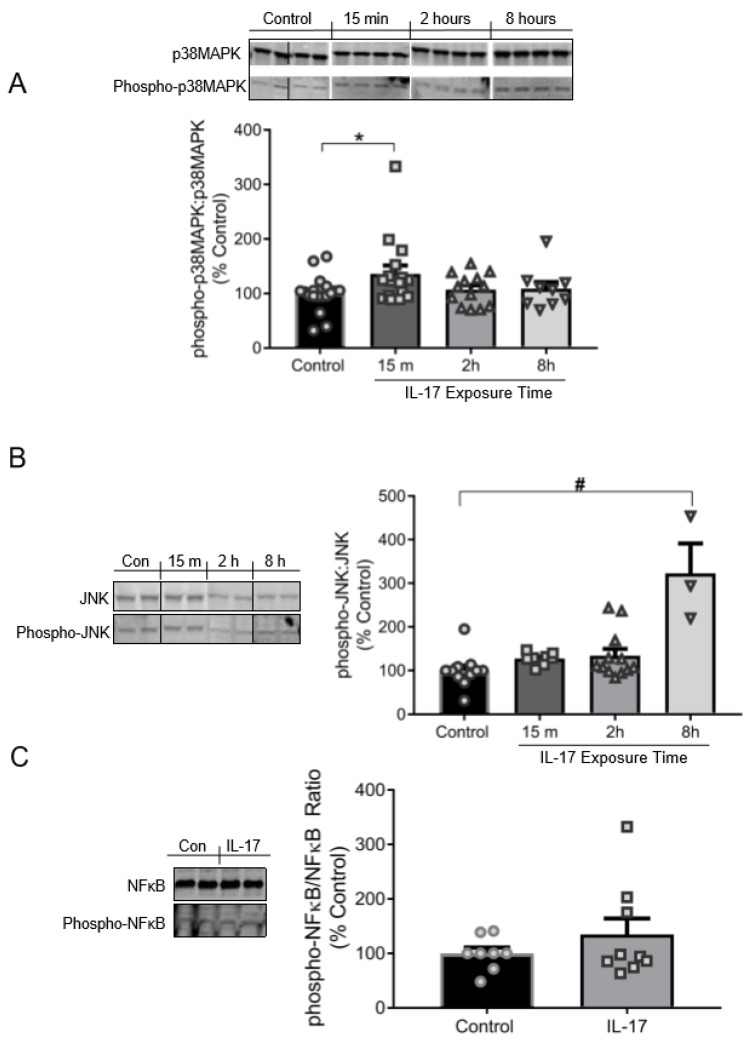
IL-17 induces phosphorylation of p38MAPK and JNK in a temporally biphasic fashion, but has no effect on NFκB in VSMCs. (**A**) Top panel: Representative immunoblots depicting p38MAPK and phospho-p38MAPK. Bottom panel: Quantification of the ratio of phospho-p38MAPK to native p38MAPK following 15 m (*n* = 16), 2 h (*n* = 13), or 8 h (*n* = 19) of IL-17 treatment (100 ng/mL) compared to control cells (*n* = 18). (**B**) Left panel: Representative immunoblots depicting JNK and phospho-JNK. Right panel: Quantification of the ratio of phospho-JNK to native JNK protein expression following 15 m (*n* = 8), 2 h (*n* = 12), or 8 h (*n* = 3) of IL-17 treatment (100 ng/mL) compared to control cells (*n* = 11). (**C**) Left panel: Representative immunoblots depicting NFκB and phospho-NFκB. Right panel: Quantification of the ratio of NFκB and phospho-NFκB protein expression in control (*n* = 8) and IL-17 (*n* = 9; 100 ng/mL) treated cells following 16 h of treatment. Images shown in each panel were acquired from the same blot under the same experimental conditions, demarcated, and arranged according to the order data is presented. Comparisons were made by one-way ANOVA, followed by Holm–Sidak post hoc test. All data are presented as mean ± SEM. * *p* < 0.05 vs. control; # *p* < 0.001 vs. control.

Exposure to IL-17 (100 ng/mL) induced phosphorylation of p38MAPK and JNK in cultured VSMCs (Figure 3A). Phospo-p38MAPK:p38MAPK was increased to 137% ± 15% of control cells (100% ± 8%) by 15 min in IL-17-treatred VSMCs (*p* = 0.0487). Phosphorylation of p38MAPK returned to baseline levels by 2–8 h, suggesting p38 is rapidly, but modestly, activated. Phospho-JNK:JNK was not significantly elevated in IL-17-treated cells until 8 h of IL-17 treatment relative to control cells (323% ± 69% vs. 100% ± 12%; *p* < 0.001; Figure 3B). The linear slope of the relationship between the % increase in JNK phosphorylation to IL-17 exposure time was +44.1 (*p* < 0.001), indicating a time-dependent response in JNK phosphorylation by IL-17. However, there was no relationship found between IL-17 exposure time and p38MAPK phosphorylation. These results identify an interesting temporal regulation of the signaling pathway: p38MAPK is transiently activated early (15 min), and JNK late (8 h). IL-17 had no significant effect on NFκB phosphorylation at 16 h of treatment compared to control cells (144% ± 33% vs. 100% ± 11%; *p* = 0.156; Figure 3C).

### 3.4. Role of p38MAPK and JNK in the Reduction of βENaC by IL-17

**Figure 4:**
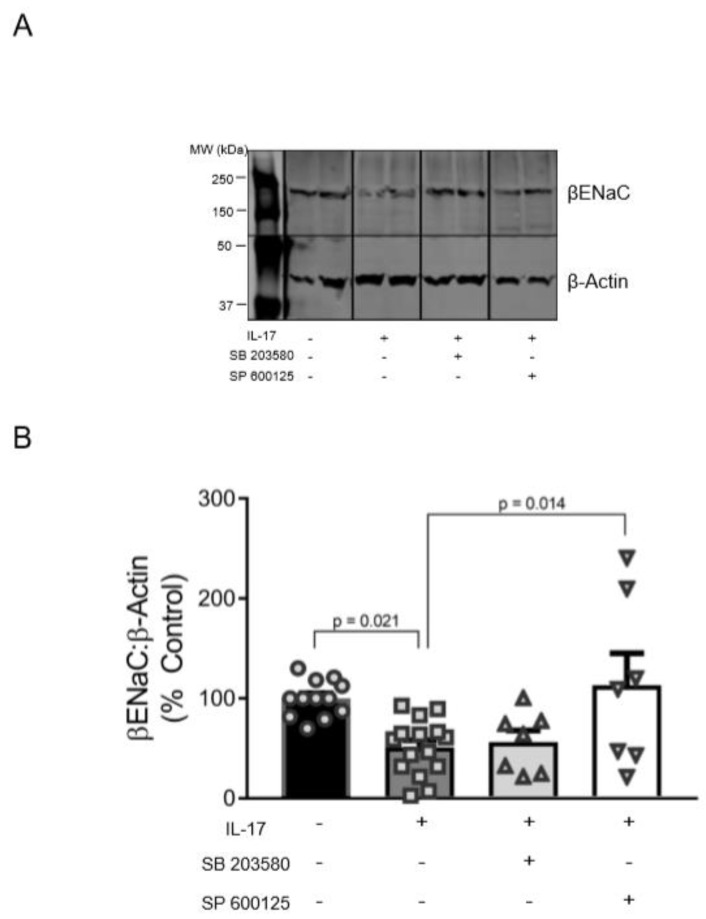
Inhibition of p38MAPK and JNK signaling pathways prevent IL-17-induced decreases in βENaC protein expression in VSMCs. VSMCs were treated for 16 h with IL-17 (100 ng/mL), with or without SB203580 (MAPK inhibitor) or SP600125 (JNK inhibitor). (**A**) Representative immunoblot depicting βENaC and β-actin control, IL-17, and IL-17 + SB203580 or SP600125. Images shown in each panel were acquired from the same blot under the same experimental conditions, demarcated, and arranged according to the order data is presented. (**B**) Quantification of βENaC in the control (*n* = 12), IL-17 (*n* = 15), IL-17 + SB203580 (*n* = 7), and IL-17 + SP600125 (*n* = 7) treated VSMCs. Comparisons were made by one-way ANOVA followed by the Holms–Sidak post hoc test. All data are presented as mean ± SEM with *p* values shown.

To examine whether p38MAPK and/or JNK pathways mediate a reduction in membrane-associated βENaC by IL-17, we treated VSMCs with 100 ng/mL IL-17 for 16 h in the presence of specific blockers to p38MAPK (SB203580) and JNK (SP600125). Representative blot and group data are shown in Figure 4A,B. IL-17 reduced βENaC to 51% ± 7% compared to untreated control cells (100% ± 5%; *p* = 0.021). JNK inhibition blocked this effect (113% ± 32% vs. 51% ± 7%, *p* = 0.014) and restored βENaC to basal levels (Figure 4B). While inhibition of p38MAPK prevented a significant decrease in βENaC by IL-17 treatment, it had little effect on βENaC protein expression, suggesting that the JNK pathway is primarily involved in IL-17-mediated βENaC reduction in VSMCs.

## 4. Discussion

The importance of ENaC proteins in epithelial Na^+^ and water transport is well established; however, a novel role for ENaC proteins as stretch sensors in VSMCs is gaining momentum [1,4,6,7,8,9,10,11,12,13,14,15,16,18,45]. This novel role is supported by the evolutionary relationship of ENaC and mechanosensing proteins in *Caenhorhabditis elegans* [1,4,5,6,7,16,19,46,47] and empirical evidence showing pressure-induced constriction, a mechanism of blood flow autoregulation, is dependent on normal VSMC βENaC expression. Since altered autoregulation is associated with end-organ injury in the kidney, which is documented in mice with reduced levels of βENaC, understanding how βENaC expression is regulated during disease processes is critical to identifying approaches to protect against loss of βENaC mediated constrictor responses [9,48]. In other words, reduced levels of βENaC in VSMCs could lead to impaired vascular function and end organ injury.

Inflammatory processes are also linked to loss of vascular function, including blood flow autoregulation [48,49]. However, the mechanisms mediating autoregulatory loss are unclear. One possibility is that certain inflammatory cytokines may inhibit expression of βENaC. A recent study from our laboratory revealed the cytokine TNF-α inhibits VSMC βENaC via MAPK pathways [50]. In epithelial cells, ENaC is also regulated by pro-inflammatory cytokines [25,26,27]. Interleukins and TNF-α have been shown to reduce ENaC through multiple cell signaling pathways [25,26,29,30]. This is the first study to examine the role and pathways mediating the pro-inflammatory cytokine IL-17 inhibition of VSMC βENaC protein expression.

Findings from this study indicate that IL-17 inhibits βENaC expression in a concentration-dependent fashion in VSMC. Pathological serum IL-17 levels range from 15–20 pg/mL, and although the concentrations used in this study are supra-physiological (50–5000 fold greater), they are consistent with other studies examining the effect of cytokine-activated signaling pathways in vitro [28,51,52,53]. The reduction in βENaC by IL-17 was not due to cell death in VSMCs. However, IL-17 treatment reduced the number of live cells, indicating a decrease in cell proliferation/viability. A decrease in βENaC could be associated with cell viability signaling pathways since there was a concentration-dependent decrease in both βENaC and cell viability by IL-17. While IL-10 and IL-19 reduce proliferation in VSMCs, this is the first study to demonstrate the anti-proliferative effect of the IL-17 in rat VSMCs [54,55,56].

Next, we observed temporal patterns in effective p38 and JNK phosphorylation by IL-17. While phosphorylation of p38 did not persist beyond 15 m, it is possible that peak p38 activation occurs prior to the 15 m time point. Conversely, JNK activation was delayed, but a time-dependent increase in phosphorylation of JNK was observed throughout the course of IL-17 treatment. This indicates that the initial upstream mechanisms involving MAPK (p38/JNK) signaling mediated by IL-17 display temporally biphasic effects. IL-17 is reported to regulate p38 and JNK pathway differentially in numerous in vitro models [57,58,59,60]. Our finding of rapid p38, but delayed JNK activation in VSMCs is consistent with the temporal differences in MAPK activation in other cells [61]. Moreover, a dynamic feed-forward/backward relationship with IL-17 and p38 has been observed, suggesting that p38 cell signaling pathways may be more responsive to IL-17 initially compared JNK [57,58]. Although IL-17 has been demonstrated to activate NFκB in several models, the phosphorylation event may not have been captured in this study following 16 h of IL-17 exposure [62,63,64]. However, IL-17 is described as a weak activator of NFκB relative to other cytokines, including TNF-α, which is consistent with our observation in this cell line [50,57]. It is possible that NFκB phosphorylation was increased at an earlier time point; however, our decision to assess NFκB phosphorylation at 16 h of IL-17 treatment was based on our previous results with TNF-α.

In comparison to IL-17, the TNF-α-mediated p38 and JNK phosphorylation persisted longer (15 min–8 h of treatment) and was more robust (~2-fold increase in phosphorylation) [50]. TNF-α also significantly increased the phosphorylation of NFκB at 16 h of treatment, indicating that not all pro-inflammatory cytokines operate through the same signaling pathways in VSMCs. Thus, it is likely that multiple cytokines, including but not limited to TNF-α and IL-17, operate together to achieve an ideal MAPK phosphorylation status to alter vascular function in certain inflammatory diseases.

Consistent with the MAPK activation patterns induced by IL-17 treatment, the reduction in βENaC was prevented by inhibiting JNK following 16 h of treatment. Due to the transient activation of p38MAPK, which returned to baseline by 2 h, it is not surprising that blocking p38MAPK did not rescue the effects of IL-17 on βENaC by 16 h. In contrast to p38MAPK, JNK phosphorylation peaked by 8 h, suggesting that delayed JNK phosphorylation may be a major driver of MAPK signaling mediated βENaC regulation in VSMCs. Because there is crosstalk among MAPK, transient activation of p38 may partially contribute to βENaC levels by escalating phosphorylation of other kinases [65]. It is possible that these pathways converge on a downstream molecular target, where JNK is a larger contributor. For example, IL-17-mediated NFκB activation was found to be blocked by p38 inhibition, and because neither NFκB nor βENaC was affected by IL-17 and p38 inhibition, respectively, it is possible that regulation of βENaC occurs through downstream effectors more responsive to JNK, such as activator protein-1 [59]. Alternatively, IL-17 has been shown to modulate mRNA stability and RNA binding proteins, leading to increased translation or degradation, which could occur through MAPK signaling [31]. IL-17 also induces reactive oxygen species in VSMCs which may also be involved in altering βENaC expression [66]. Further studies are necessary to fully understand how multiple pro-inflammatory cytokines may interact to navigate downstream events involved in βENaC regulation by IL-17.

This study highlights the role of IL-17 in regulating membrane-associated βENaC expression in VSMC, which holds important implications for pro-inflammatory vascular diseases. Understanding the components involved in mediating membrane-associated expression of βENaC by inflammatory factors could unveil potential pharmacotherapeutic targets to ameliorate vascular health during inflammation.

## 5. Conclusions

Numerous lines of evidence suggest βENaC is a mechanosensor in VSMCs where it contributes to pressure-induced tone and protection against end-organ injury. Findings from this study suggest pressure-induced autoregulation of blood flow impairment in certain inflammation associated disorders may be due to elevated pro-inflammatory cytokines inhibition of VSMC βENaC, leading to subsequent vascular/organ injury. For example, placental ischemia increases circulating IL-17, impairs cerebral myogenic tone and CBF autoregulation, and also reduces cerebrovascular and placental βENaC [67,68,69,70,71,72,73]. This study demonstrates that IL-17 directly reduces βENaC in VSMCs, indicating that IL-17 may contribute to impaired vascular function in pro-inflammatory disorders, such as preeclampsia. Future studies are planned to address this point.

## Supplementary Materials

The following are available online at https://www.mdpi.com/1422-0067/21/8/2953/s1, Figure S1. Western blot detection of epitope tagged _HA_mbENaCV5 in COS-7 and A10 cells.
