# Supplementary material for: Interleukin-17 Reduces βENaC via MAPK Signaling in Vascular Smooth Muscle Cells
Source: Int J Mol Sci. 2020 Apr 22;21(8):2953. doi: 10.3390/ijms21082953 (PMC7215799; doi:10.3390/ijms21082953)
Supplement: Supplementary file 1 [file ijms-21-02953-s001.pdf]

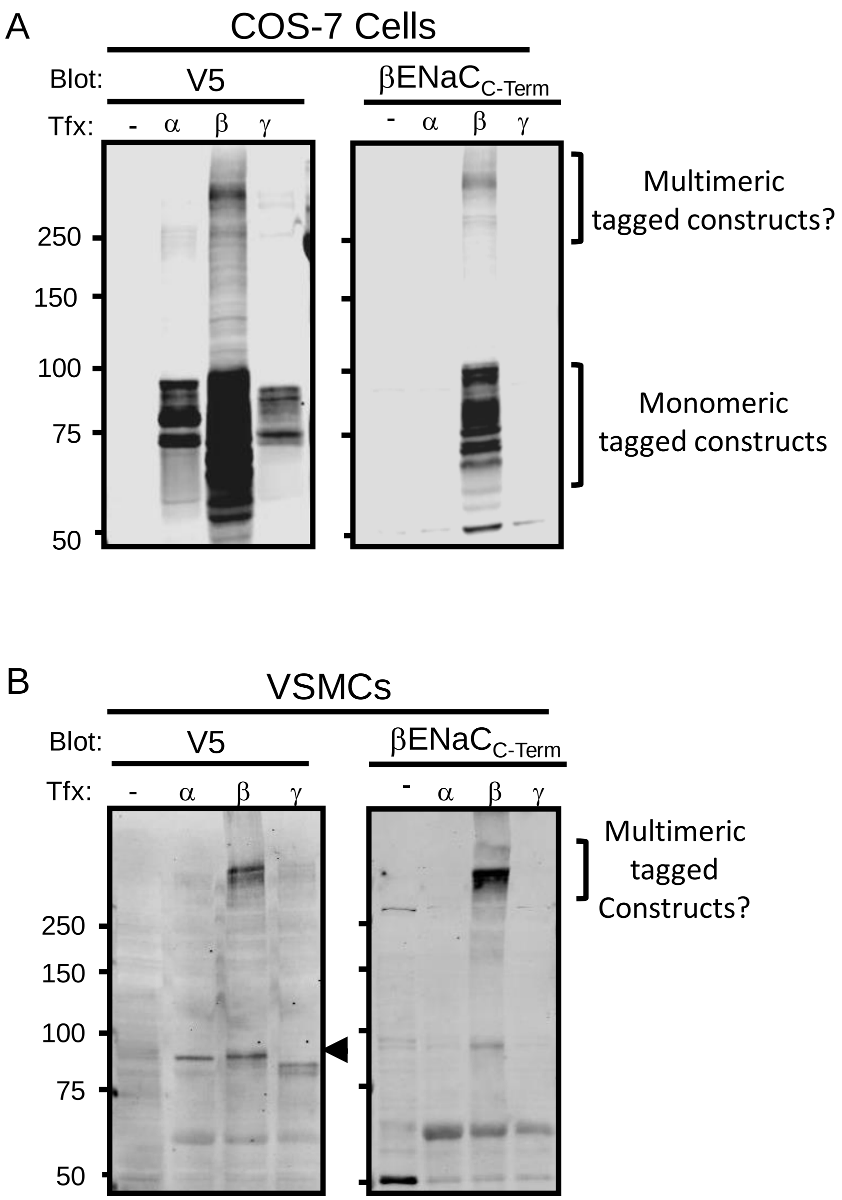

Figure S1. Western blot detection of epitope tagged $_{HA}$mbENaCV5 in COS-7 and A10 cells. Epitope tagged mouse bENaC construct ($_{HA}$bENaC$_{V5}$) generously provided to our laboratory by Dr. Thomas Kleyman is robustly expressed in COS-7 cells and migrates as expected with glycosylation. The panel at right demonstrates our antibody specifically labels bENaC as well as anti-V5. B. The same construct expresses weakly in A10 cells, due to the lack of SV-40 promoter in A10 cells, however, bENaC construct is expressed at a much higher molecular weight. The molecular masses are slightly different in this figure because we are detecting an exogenous, tagged construct and examining expression in crude, whole cell lysates.
